# Analysis benefits of a second Allo-HSCT after CAR-T cell therapy in patients with relapsed/refractory B-cell acute lymphoblastic leukemia who relapsed after transplant

**DOI:** 10.3389/fimmu.2023.1191382

**Published:** 2023-07-04

**Authors:** Xing-yu Cao, Jian-ping Zhang, Yan-li Zhao, Min Xiong, Jia-rui Zhou, Yue Lu, Rui-juan Sun, Zhi-jie Wei, De-yan Liu, Xian Zhang, Jun-fang Yang, Peihua Lu

**Affiliations:** ^1^ Department of Bone Marrow Transplantation, Hebei Yanda Lu Daopei Hospital, Langfang, China; ^2^ Department of Hematology, Hebei Yanda Lu Daopei Hospital, Langfang, China; ^3^ Beijing Lu Daopei Institute of Hematology, Beijing, China

**Keywords:** CAR-T, B-ALL, relapse, second transplant, LFS, NRM

## Abstract

**Background:**

Chimeric antigen receptor (CAR) T-cell therapy has demonstrated high initial complete remission (CR) rates in B-cell acute lymphoblastic leukemia (B-ALL) patients, including those who relapsed after transplant. However, the duration of remission requires improvements. Whether bridging to a second allogeneic hematopoietic stem cell transplant (allo-HSCT) after CAR-T therapy can improve long-term survival remains controversial. We retrospectively analyzed long-term follow-up data of B-ALL patients who relapsed post-transplant and received CAR-T therapy followed by consolidation second allo-HSCT to investigate whether such a treatment sequence could improve long-term survival.

**Methods:**

A single-center, retrospective study was performed between October 2017 and March 2022, involving 95 patients who received a consolidation second transplant after achieving CR from CAR-T therapy.

**Results:**

The median age of patients was 22.8 years (range: 3.3-52.8) at the second transplant. After the first transplant, 71 patients (74.7%) experienced bone marrow relapse, 16 patients (16.8%) had extramedullary relapse, 5 patients (5.3%) had both bone marrow and extramedullary relapse and 3/95 patients (3.2%) had positive minimal residual disease (MRD) only. Patients received autologous (n=57, 60.0%) or allogeneic (n=28, 29.5%) CAR-T cells, while 10 patients (10.5%) were unknown. All patients achieved CR after CAR-T therapy. Before second HSCT, 86 patients (90.5%) were MRD-negative, and 9 (9.5%) were MRD-positive. All second transplant donors were different from the first transplant donors. The median follow-up time was 623 days (range: 33-1901) after the second HSCT. The 3-year overall survival (OS) and leukemia-free survival (LFS) were 55.3% (95%CI, 44.3-66.1%) and 49.8% (95%CI, 38.7-60.9%), respectively. The 3-year relapse incidence (RI) and non-relapse mortality (NRM) were 10.5% (95%CI, 5.6-19.6%) and 43.6% (95%CI, 33.9-56.2%), respectively. In multivariate analysis, the interval from CAR-T to second HSCT ≤90 days was associated with superior LFS(HR, 4.10, 95%CI,1.64-10.24; *p*=0.003) and OS(HR, 2.67, 95%CI, 1.24-5.74, *p*=0.012), as well as reduced NRM (HR, 2.45, 95%CI, 1.14-5.24, *p*=0.021).

**Conclusions:**

Our study indicated that CAR-T therapy followed by consolidation second transplant could significantly improve long-term survival in B-ALL patients who relapsed post-transplant. The second transplant should be considered in suitable patients and is recommended to be performed within 90 days after CAR-T treatment.

## Introduction

Relapse following allogeneic hematopoietic stem cell transplant (allo-HSCT) remains the major cause of post-transplant mortality in patients with B-cell acute lymphoblastic leukemia (B-ALL) (1, 2). Traditional treatments, such as chemotherapy, donor lymphocyte infusion (DLI) and second transplant for post-transplant relapse demonstrated relatively poor prognoses (3, 4). In recent years, immunotherapeutic agents like blinatumomab and inotuaumab ozogamicin have shown potential in increasing response rates and overall survival (OS) in refractory and relapsed(R/R) B-ALL. However, in B-ALL patients relapsed after allo-HSCT and treated with blinatumomab, the 1-year OS was only 36% and 18% at 3 years, even after achieving complete remission (CR) (5). Likewise, long-term survival proved difficult for inotuaumab ozogamicin-treated post-transplant relapsed B-ALL patients, with a median relapse-free survival of 12.0 months (6). Additionally, blinatumomab and inotuaumab ozogamicin exhibited limited efficacy in B-ALL with extramedullary disease (EMD) (7, 8).

Chimeric antigen receptor (CAR)s are designed to bind to specific antigens, activating CAR-T cells without the conventional dual restriction imposed by specific T cell receptor and the major histocompatibility complex (MHC) (9). Patients with HLA loss and HLA antigen downregulation after allo-HSCT may also benefit from CAR-T therapy (10). In B-ALL patients who relapsed after allo-HSCT, the initial CR rate following CAR-T cell infusion can reach about 85.7%~93.8% (11–14). CAR-T has also proven effective in treating patients with extramedullary lesions (15). However, the duration of remission requires significant improvement (16). The question of whether bridging to a second allo-HSCT after CAR-T therapy can enhance long-term survival for B-ALL patients who relapsed after allo-HSCT remains disputed. In this study, we retrospectively analyzed the long-term follow-up data of B-ALL patients who experienced relapse after their first transplant and underwent CAR-T therapy, followed by consolidation second allo-HSCT. Our aim was to determine whether this treatment sequence could improve long-term survival.

## Materials and methods

### Study design and patient enrollment

We conducted a retrospective study analyzing patients with B-ALL who underwent a second allo-HSCT at Hebei Yanda Lu Daopei Hospital between October 18, 2017, and March 4, 2022. To be eligible for enrollment, patients had to meet the following criteria (1): morphological and/or extramedullary relapse or minimal residual disease (MRD)-positive after the first HSCT (2), received CAR-T therapy relapsed or MRD-positive after the first transplant and (3) received a second allogeneic HSCT at our center. The last follow-up date was January 1, 2023. Since most patients’ first transplants were performed at other centers, information on the first transplant was obtained from medical records. CAR-T therapy before the second transplant could be performed at our center or other centers, with data collected from medical records or clinical trials registered at www.chictr.org.cn ChiCTR2000031340, ChiCTR-ONC-17012829, ChiCTR1800016541, ChiCTR1800017439, ChiCTR2000038532, and www.clinicaltrials.gov NCT04100187, NCT02546739, NCT03312205, NCT03825718, NCT05225831, NCT03825731, NCT03173417, NCT04163575, NCT03953599, NCT04260945, NCT04792593 (15). All second transplants were performed at our transplant center, providing detailed information. The study was approved by the Ethics Committee of Hebei Yanda Lu Daopei Hospital. The primary endpoints were leukemia-free survival (LFS) and overall survival (OS) after the second transplant. Secondary endpoints included the cumulative incidences of acute and chronic graft-versus-host disease (GVHD), non-relapse mortality (NRM), and relapse incidence (RI).

### Lymphodepleting chemotherapy and CAR-T cell therapy

Some patients have received more than once CAR-T treatment after relapse or MRD-positive after the first transplant. This study focused on analyzing the impact of the last CAR-T cell infusion before the second transplant on the outcome of the second transplant. Unless otherwise stated, CAR-T in this study refers to the last CAR-T prior to the second transplant. Patients received fludarabine (30 mg/m^2^/day) and cyclophosphamide(250 mg/m^2^/day) (FC) lymphodepleting chemotherapy for three consecutive days prior to CAR-T cell infusion(Day-5 ~ Day-3) (15). Anti-tumor response was initially assessed on day 15 or day 30 after CAR-T cell infusion. Bone marrow specimens are used to evaluate MRD levels, while patients with EMD underwent positron emission tomography-computed tomography (PET-CT) or magnetic resonance imaging (MRI) on Day 30. Complications such as cytokine release syndrome(CRS), immune effector cell-associated neurotoxicity syndrome(ICANS) and GVHD were assessed after CAR-T therapy. The identification, evaluation, and management of CRS and ICANS after CAR-T cell therapy were in accordance with the ASCO guideline (17). CAR-T was classified into autologous CAR-T and allogeneic CAR-T according to the source of T cells for CAR-T cell production. T lymphocytes for autologous CAR-T and allogeneic CAR-T were isolated from the patient’s and donor’s peripheral blood mononuclear cells (PBMCs), respectively. Patients received CAR-T cells that target either CD19, CD22 or CD20. The costimulatory domains of CAR-T were CD28, 4-1BB and OX40. The majority of CAR-T treatments utilized murine single-chain variable fragments (scFvs), while a small number employed humanized scFvs.

### Laboratory tests related to second HSCT

High-resolution HLA-A, B, Cw, DRB1, and DQ-loci were obtained for all second HSCT recipients and their donors. MRD was detected using quantitative polymerase chain reaction (qPCR) to identify leukemia-related fusion genes and multiparameter flow cytometry (FCM) to detect leukemia-associated immunophenotypes (LAIP). The minimum sensitivities for monitoring MRD were 1×10^−5^ for gene markers and 1×10^−3^ to 1×10^−4^ for LAIPs. Plasma cytomegalovirus (CMV) DNA and Epstein-Barr virus (EBV) DNA were detected and quantified using qPCR up to 100 days post-transplant.

### Conditioning regimen in second HSCT

Patients received total body irradiation/cyclophosphamide (TBI/Cy), TBI/fludarabine (TBI/Flu), busulfan/cyclophosphamide (Bu/Cy) or busulfan/fludarabine (Bu/Flu) based conditioning regimens. TBI/Cy regimens included cytarabine (2-3g/m^2^/d IV, days -10 and -9), TBI (total dose 8-12Gy), cyclophosphamide (1.8g/m^2^/d IV, days -5 and -4), and simustine (250mg/m^2^ oral, day -3). TBI/Flu regimens comprised cytarabine (2-3g/m^2^/d IV, days -10 and -9), TBI (total dose was 8-12Gy), fludarabine (30mg/m^2^/d IV, days -6 and -2), and simustine (250mg/m^2^ oral, day -3). Bu/Cy regimens consisted of cytarabine (2-3g/m^2^/d IV, days -11 and -10), Bu (3.2mg/kg/d IV, days -9 to -6), cyclophosphamide (1.8g/m^2^/d IV, days -5 and -4), and simustine (250mg/m^2^oral, day -3). Bu/Flu regimens involved cytarabine (2-3g/m^2^/d IV, days -11 and -10), Bu (3.2mg/kg/d IV, days -9 to -6), fludarabine (30mg/m^2^/d IV, days -6 and -2), and simustine (250mg/m^2^ oral, day -3). The intensified conditioning regimens included the addition of thiotepa (5mg/kg×1-2days,IV), etoposide (15mg/kg×1-2days or 30mg/kg×1day, IV) or FLAG (fludarabine 30mg/m^2^×5days; cytarabine 2g/m^2^×5days; G-CSF 5μg/kg/day×5days) to one of the standard regimens.

### GVHD prophylaxis in second HSCT

For GVHD prophylaxis, anti-human T lymphocyte immunoglobulin (ATG), calcineurin inhibitors (tacrolimus or cyclosporine), mycophenolate mofetil, and intravenous methotrexate (15 mg/m^2^ on day +1, then 10 mg/m^2^ on days +3, +6, and +11) were utilized. The ATGs applied in conditioning regimens included ATG-F (formerly Fresenius, now Grafalon, Neovii Biotech GmbH), ATG-T (Thymoglobulin, Genzyme Polyclonals S.A.S.), and ATG-P (anti-human T lymphocyte porcine immunoglobulin, Wuhan Institute of Biological Products Co., Ltd.). The total dose of ATG-F was 20mg/kg. The total dose of ATG-T was 5mg/kg, 6mg/kg, 7mg/kg, 7.5mg/kg or 8.5mg/kg, respectively. The total dose of ATG-P was 80 mg/kg. The total ATG dose was administered as an intravenous infusion, divided over four days from day -2 to day -5.

### Mobilization, collection, and infusion of grafts

G-CSF at a dose of 5μg/kg q12h was administered to the donors on days -5, -4, -3, -2, and -1. For matched sibling donor (MSD)-HSCT and haploidentical HSCT (haplo-HSCT), bone marrow stem cells were collected on day 01 and peripheral stem cells were used on day 02. For unrelated donor (URD)-HSCT, only peripheral stem cells were used. G-CSF mobilized fresh bone marrow and peripheral blood stem cell grafts were infused.

### Definitions and follow-up

The influence of various pre-transplant comorbidities was assessed using the hematopoietic cell transplantation specific comorbidity-index (HCT-CI) (18). Neutrophil engraftment was defined as the first 3 consecutive days with absolute neutrophil count (ANC) ≥0.5×10^9^/L. Platelet engraftment was defined as the first 7 consecutive days with platelet counts ≥20×10^9^/L without infusion. Acute GVHD (aGVHD) was diagnosed and graded according to previously published consensus criteria (19, 20). Chronic GVHD (cGVHD) was defined and graded according to the National Institutes of Health consensus criteria. CR was defined as bone marrow blasts <5%, absence of circulating blasts and extramedullary disease, ANC≥1.0×10^9^/L and platelet count≥100×10^9^/L. CR with incomplete hematologic recovery (CRi) was defined as meeting all CR criteria except for neutropenia (<1.0×10^9^/L) or thrombocytopenia (<100×10^9^/L) (21). Transplant-associated thrombotic microangiopathy (TA-TMA) was diagnosed according to published diagnostic criteria proposed by Jodele et al (22). MRD-negative was defined as CR with negativity by qPCR and multiparameter FCM. OS was defined as the time from the first day of the second transplant to death from any cause or the last follow-up. LFS was defined as the time from second transplant to relapse or death, or last follow-up. RI was defined as the reappearance of blasts in the blood, bone marrow (>5%), or any extramedullary site after achieving CR. NRM was defined as death without relapse.

### Statistical analysis

Outcomes including LFS, OS, GVHD incidence, NRM and RI were measured from the time of stem cell infusion in second transplant. OS and LFS were analyzed by the Kaplan-Meier method using the log-rank test. Cumulative incidence of NRM, relapse, CMV, EBV, aGVHD and cGVHD were performed using Gray’s test, with death before the event of interest as a competing risk. Factors with a 2-sided *p* value ≤0.15 in a univariate analysis were subjected to multivariate analysis. A 2-sided *p*<0.05 was considered statistically significant. Results were reported as the hazard ratio (HR) with 95% confidence interval(CI). NCSS12 and R3.4.4 statistical software were used for statistical analysis.

## Results

### Patient characteristics

A total of 99 consecutive pediatric and adult patients with B-ALL were screened in this study, who relapsed or became MRD-positive after the first transplant and were treated with CAR-T followed by a consolidation second transplant at our center. Four patients were excluded because they did not achieve remission after CAR-T therapy. Finally, 95 B-ALL patients were enrolled and analyzed in this retrospective study. Patients had a median age of 21.2 years (range: 2.7-51.7). Detailed patient characteristics at first HSCT are shown in **Table 1**. The median time from diagnosis to first transplant was 244 (range: 109-2766) days, while the median time from diagnosis to second transplant was 840 (range: 313-4486) days. The median interval between the first and second transplant was 518 (range: 160-4377) days. Ninety-three of the first HSCT patients underwent allo-HSCT and the other 2 underwent autologous HSCT (auto-HSCT). After their first transplant, 34 patients (35.8%) initially became MRD-positive, with three remaining MRD-positive and 31 eventually developing bone marrow or extramedullary relapse. Sixty-one patients (64.2%) experienced direct bone marrow and/or extramedullary relapse. Among them, 71 (74.7%) had bone marrow relapse, 5 (5.3%) had bone marrow and extramedullary relapse, and 16 (16.8%) had extramedullary relapse. The median time to MRD positivity after the first transplant was 139 days (range: 29-776 days), while the median time to bone marrow or extramedullary relapse was 314 days (range: 64-4121 days). The median time from MRD-positive to bone marrow or extramedullary relapse was 112 days (range: 18-2442 days). Patients who developed positive MRD after their first transplant received treatments such as post-immunosuppressive dose reduction, tyrosine kinase inhibitor (TKI) drugs, and DLI. The treatments received by patients after MRD-positive or relapse are detailed in **Table S1**. Three patients with persistent MRD-positive received CAR-T cell therapy directly. Patients who relapsed after bone marrow and/or extramedullary treatment received therapies such as direct CAR-T therapy, chemotherapy combined with CAR-T therapy, or chemotherapy combined with DLI followed by CAR-T therapy.

**Table 1:** Patient characteristics at first transplant (n=95).

Characteristics	
Age of patients at first HSCT, yr, median (range)	21.2 (2.7-51.7)
Gender, n (%)	
Male	56 (59.0%)
Female	39 (41.1%)
Fusion genes at diagnosis, n (%)	
BCR::ABL1	21 (22.1%)
E2A::HLF	2 (2.1%)
E2A::PBX1	5 (5.3)
EBF1::PDGFRB	2 (2.1%)
EP300::ZNF384	1 (1.1%)
IGH::EPOP/MILT3::CISD3	1 (1.1%)
MLL::AF4	2 (2.1%)
NUP214::ABL1	2 (2.1%)
PICALM::MLLT10	1 (1.1%)
TAF15::ZNF384	1 (1.1%)
TEL::AML1	4 (4.2%)
TERP2::JAK2	1 (1.1%)
Negative	52 (54.7%)
First HSCT type, n (%)	
Matched sibling donor	30 (31.6%)
Matched-unrelated donor	11 (11.6%)
Haploidentical donor	49 (51.6%)
Autologous	2 (2.1%)
Cord blood transplant	3 (3.2%)
MRD status pre-first HSCT, n (%)	
MRD-negative	61 (64.2%)
MRD-positive	32 (33.7%)
Not available	2 (2.1%)
Disease status pre-first HSCT; n (%)	
CR1	65 (68.4%)
CR2	23 (24.2%)
CR3	4 (4.2%)
NR	3 (3.2%)
ABO compatibility, n (%)	
Matched	34 (35.8%)
Major mismatched	14 (14.7%)
Minor mismatched	11 (11.6%)
Bidirectional mismatched	4 (4.2%)
Not available	32 (33.7%)
Conditioning regimen of first HSCT, n (%)	
Bu-based	64 (67.4%)
TBI-based	19 (20.0%)
Mel-based	1 (1.1%)
Not available	11 (11.6%)
Sources of hematopoietic stem cells, n (%)	
Bone marrow and peripheral blood	37 (39.0%)
Peripheral blood	33 (34.7%)
Cord blood	3 (3.2%)
Not available	22 (23.2%)
Site of final relapse after first HSCT, n (%)	
Bone marrow	71 (74.7%)
Bone marrow and extramedullary site	5 (5.3%)
Extramedullary site	16 (16.8%)
Only MRD-positive	3 (3.2%)

MRD, minimal residual disease; CR, complete remission; NR, no remission; Bu, busulfan; TBI, total body irradiation; Mel, melphalan; HSCT, hematopoietic stem cell transplant.

### CAR-T therapy after first HSCT relapse/MRD-positive

All 95 patients achieved CR/CRi after CAR-T therapy and underwent consolidation second allo-HSCT. Patients received a median of 1 (range: 1-5) CAR-T treatments between the first and second transplants. The median time from relapse or MRD-positive status after the first transplant to the last CAR-T cell infusion was 57 days (range: 13-1212 days). The median time between the last CAR-T therapy and the second transplant was 64 days (range: 35-246 days). Fifty-seven patients received autologous CAR-T, 28 patients received allogeneic CAR-T, and 10 patients had unavailable data. The median bone marrow blast percentage before CAR-T therapy was 23.5% (range: 0-97.6%). After CAR-T therapy, 56 (58.9%) patients developed CRS, and 8 patients had unavailable CRS data. Forty-six (48.4%) patients experienced grade 1 CRS, 7 (7.3%) patients had grade 2 CRS, 1 (1.1%) patient had grade 3 CRS, 1 (1.1%) patient had grade 4 CRS, and 1 (1.8%) patient’s CRS grade was undetermined. Five patients (5.2%) had grade 1 ICANS, and 1 patient had grade 3 ICANS after CAR-T cell therapy. Only 2 patients developed GVHD following CAR-T therapy.

### Consolidation second allo-HSCT

The median age of patients at the second transplant was 22.8 years (range: 3.3-52.8 years). HCT-CI scores, conditioning regimens, donor types, and stem cell sources are presented in **Table 2**. Nine patients (9.5%) were MRD-positive CR, and the remaining patients were MRD-negative CR before the second transplant. Besides, 66.7% (6/9) of MRD-positive patients and 15.1% (13/86) of MRD-negative patients received intensified conditioning regimens (*p*<0.001). All patients received different donors for the second transplant. Sixty-eight patients (71.6%) underwent haploidentical allo-HSCT. The median time for neutrophil and platelet engraftment was 14 days (range: 8-26 days) and 16 days (range: 6-420 days), respectively. The 30-day cumulative incidence of neutrophil engraftment was 100%. The 30-day cumulative incidence of platelet engraftment was 79.0% (95% CI, 71.2-87.6%), while the 60-day cumulative incidence of platelet engraftment was 86.3% (95% CI, 79.7-93.5%).

**Table 2:** Patient characteristics at second transplant (n=95).

Characteristics	
Age of patients at second HSCT, yr, median(range)	22.8 (3.3-52.8)
HCT-CI scores before second HSCT, n (%)	
0	47 (49.5%)
1	36 (37.9%)
2	9 (9.5%)
3	2 (2.1%)
4	1 (1.1%)
Second HSCT donor type, n (%)	
Matched sibling donor	1 (1.1%)
Matched-unrelated donor	26 (27.4%)
Haploidentical	68 (71.6%)
Changing donors in second HSCT, n (%)	
Yes	95 (100%)
No	0 (0%)
Cell sources of CAR-T, n (%)	
Autologous	57 (60.0%)
Allogeneic	28 (29.5%)
Unknown (received CAR-T therapy outside our hospital)	10 (10.5%)
Targets of CAR-T, n (%)	
CD19	79 (83.2%)
CD22	5 (5.3%)
CD19+CD22	10 (10.5%)
CD19+CD20	1 (1.1%)
MRD status pre-second HSCT, n (%)	
MRD-negative	86 (90.5%)
MRD-positive	9 (9.5%)
Disease status pre-HSCT; n (%)	
CR	95 (100%)
ABO compatibility, n (%)	
Matched	39 (41.1%)
Major mismatched	23 (24.2%)
Minor mismatched	23 (24.2%)
Bidirectional	10 (10.5%)
Conditioning regimen of second HSCT, n (%)	
Bu-based	19 (20%)
TBI-based	75 (79.0%)
Mel-based	1 (1.1%)
Conditioning regimen from first to second HSCT, n (%)	
Bu-Mel	1 (1.1%)
Bu-TBI	63 (66.3%)
Mel-TBI	1 (1.1%)
TBI-Bu	18 (19.0%)
TBI-TBI	1 (1.1%)
Not available	11 (11.6%)
Sources of hematopoietic stem cells, n (%)	
Bone marrow and peripheral blood	68 (71.6%)
Peripheral blood	27 (28.4%)
GVHD prophylaxis, n (%)	
Cyclosporine+MMF+MTX	83 (87.4%)
Tacrolimus+MMF+MTX	12 (12.6%)
Antithymocyte globulin (ATG), n (%)	
ATG-T	36 (38.0%)
ATG-F	52 (54.7%)
ATG-P	7 (7.4%)
Number of infused MNC; median(range)×10^8^/kg	8.6 (3.9-23.9)
Number of infused CD34+ cells; median(range)×10^6^/kg	5.0 (0.9-17.3)
Number of infused CD3+ cells; median(range)×10^8^/kg	1.8 (0.5-6.6)

HCT-CI, hematopoietic cell transplantation-comorbidity index; HSCT, hematopoietic stem cell transplantation; MRD, minimal residual disease; CR, complete remission; CMV, cytomegalovirus; EBV, Epstein-Barr virus; Bu, busulfan; TBI, total body irradiation; Mel, melphalan; ATG-T, ATG-thymoglobuline; ATG-F, ATG-Fresenius; ATG-P, ATG-porcine.

### GVHD

**Figure 1:**
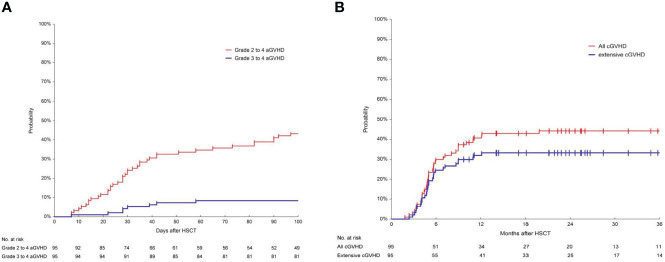
Cumulative incidence of aGVHD and cGVHD. **(A)** Cumulative incidence of grade 2-4 and grade 3-4 acute graft-versus-host disease (aGVHD) in 95 patients. The 100-day incidence of grade 2 to 4 aGVHD was 43.2% (95%CI, 34.3%-54.4%) and the incidence of grade 3 to 4 aGVHD was 8.4% (95%CI, 4.3%-16.3%). **(B)** Cumulative incidence of chronic graft-versus-host disease (cGVHD) in 95 patients. The incidence of any grade cGVHD at 180 days and at 2-year was 29.8% (95%CI, 21.8%-40.6%) and 44.2% (95CI, 35.1%-55.6%). The 180-day and 2-year cumulative incidence of extensive cGVHD was 24.5% (95%CI, 17.2%-34.9%) and 33.1% (95%CI, 24.9%-44.2%), respectively.

The 100-day incidence of grade 2 to 4 aGVHD was 43.2% (95%CI, 34.3%-54.4%) and the incidence of grade 3 to 4 aGVHD was 8.4% (95%CI, 4.3%-16.3%) (**Figure 1A**). The 180-day and 2-year cumulative incidence of any grade cGVHD was 29.8% (95%CI, 21.8%-40.6%) and 44.2% (95CI, 35.1%-55.6%), respectively. The 180-day and 2-year cumulative incidence of extensive cGVHD were 24.5% (95%CI, 17.2%-34.9%) and 33.1% (95%CI, 24.9%-44.2%) (**Figure 1B**).

### Viral infection-related complications and adverse events

The incidence of CMV viremia and EBV viremia at 100 days after second HSCT was 72.6% (95%CI, 64.2%-82.2%) and 19.0% (95%CI, 12.5%-28.7%), respectively. The incidence of post-transplant lymphoproliferative disorders (PTLD) at 100 days post-transplant was 4.2% (95%CI, 1.6%-11.0%). The cumulative incidence of TA-TMA was 8.4% (95%CI, 4.3%-16.4%) at 100 days, 14.8% (95%CI, 9.1%-24.1%) at 1 year and 19.5% (95%CI, 12.9%-29.6%) at 3 years after second HSCT.

### Cause of death

A total of 40 patients died. Causes of death included GVHD (n=13, 32.5%), infection (n=15, 37.5%), TA-TMA (n=5, 12.5%), TA-TMA and GVHD (n=1, 2.5%), TA-TMA and infection (n=1, 2.5%), TA-TMA and VOD (n=1, 2.5%), relapse (n=1, 2.5%), diffuse alveolar hemorrhage (n=1, 2.5%), Cerebral hemorrhage (n=1, 2.5%) and sudden death (n=1, 2.5%). Of the 14 patients who died from GVHD, 3 had DLI-related GVHD (2 for relapse prevention and 1 for MRD-positive treatment intervention after the second transplant), and 2 had GVHD following CD19 and/or CD22 CAR-T therapy (MRD-positive post second transplant). One case developed GVHD after anti-PD1 antibody administration following MRD-positive post second transplant. Two patients developed GVHD after the third transplant who relapsed after the second transplant and underwent a third transplant.

### LFS, OS, NRM and RI

**Figure 2:**
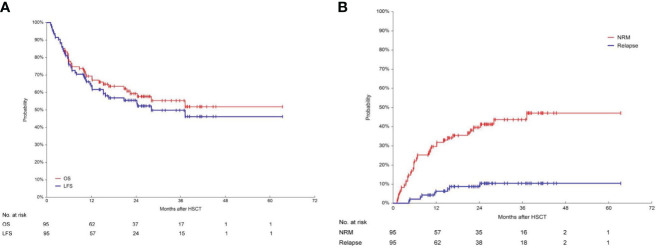
Long-term survival, including OS, LFS, RI and NRM. **(A)** Overall survival (OS) and leukemia-free survival (LFS) in 95 patients. After the second transplant, the 1-year OS and LFS were 69.3% (95%CI, 59.7%-78.2%) and 63.9% (95%CI, 54.0%-73.3%) respectively. The 3-year OS and LFS were 55.3% (95%CI, 44.3%-66.1%) and 49.8% (95%CI, 38.7%-60.9%), respectively. **(B)** Relapse incidence (RI) and non-relapse mortality (NRM) in 95 patients. The 3-year RI was 10.5% (95%CI, 5.6%-19.6%). The 3-year NRM was 43.6% (95%CI, 33.9%-56.2%).

The median follow-up time for patients after second transplant was 623 days (range: 33-1901). After the second transplant, the 1-year OS and LFS were as high as 69.3% (95%CI, 59.7%-78.2%) and 63.9% (95%CI, 54.0%-73.3%), respectively (**Figure 2A**). The 1-year RI was 6.5% (95%CI, 3.0%-14.0%) and the 1-year NRM was 30.7% (95%CI, 22.7%-41.6%) (**Figure 2B**). The 3-year OS and LFS reached 55.3% (95%CI, 44.3%-66.1%) and 49.8% (95%CI, 38.7%-60.9%), respectively. The 3-year RI was 10.5% (95%CI, 5.6%-19.6%) and the 3-year NRM was 43.6% (95%CI, 33.9%-56.2%).

### Univariate analysis

Sex, age, BCR::ABL1 fusion gene, time to relapse after the first transplant (≤180 days and >180 days), interval between the first and second transplant, interval between the last CAR-T infusion time and the second transplant, cell origin of the CAR-T (autologous and allogeneic), occurrence of CRS after CAR-T, MRD status before the second transplant, conditioning regimens based on TBI or busulfan for the second transplant, the type of ATG of the second transplant were statistically analyzed as influencing factors. Our previous published data showed that the median time to relapse after CD19 CAR-T therapy for B-ALL patients was 100 days (11). In this study, the median time between the last CAR-T therapy and the second transplant was 64 days (range: 35-246). In view of this, we also compared the prognosis between the two groups of patients with an interval of ≤90 days vs >90 days and ≤60 days vs >60 days, respectively. As shown in **Table S2**, there were no differences in baseline characteristics between the ≤90 days and >90 days groups. When comparing the two groups of patients with ≤90 days and >90 days between the last CAR-T infusion and the second transplant, significant differences were observed in LFS, OS, and NRM (3-year LFS 53.9% [95%CI, 41.8% to 65.8%] vs 18.2% [95%CI, 2.1% to 45.1%], *p*=0.001;3-year OS 58.9% [95%CI, 46.8% to 70.4%] vs 27.3% [95%CI, 6.3% to 55.9%], *p*=0.003; 3-year NRM 41.2% [95%CI, 30.8%-55.0%] vs 63.6% [95%CI, 40.7% to 99.5%], *p*=0.030, yet no difference in RI (3-year RI 9.5% [95%CI, 4.6% to 19.4%] vs 18.2% [95%CI, 5.2% to 63.7%], *p*=0.342). However, the differences in OS, LFS, NRM and RI were not statistically significant between the two groups with the interval ≤60 days and >60 days. In univariate analysis, the factors influencing LFS were BCR::ABL1, interval from CAR-T to second HSCT, type of CAR-T, type of ATG (**Table 3**). The factors affecting OS were BCR::ABL1, and intervals from CAR-T to second HSCT. The factor influencing RI was type of ATG. The factors affecting NRM were BCR::ABL1 and interval from CAR-T to second HSCT.

**Table 3:** Univariate analyses of risk factors affecting leukemia free survival (LFS), overall survival (OS), cumulative relapse incidence (RI), and cumulative incidence of non-relapse mortality (NRM).

Factors	Events	3-year LFS % (95%CI)	*P value*	3-year OS % (95%CI)	*P* value	3-year RI % (95%CI)	*P* value	3-year NRM % (95%CI)	*P value*
**Patient gender**			0.382		0.272		0.877		0.399
Male	56	52.2 (37.8-66.5)%		61.1 (47.0-74.2)%		10.8 (4.6-25.3)%		38.9 (27.3-55.5)%	
Female	39	46.4 (30.2-63.0)%		48.4 (32.0-65.0)%		10.4 (4.1-26.3)%		49.0 (34.8-69.1)%	
**Patient age**			0.956		0.757		0.799		0.840
≤14 years	23	48.7 (27.8-69.8)%		58.6 (37.5-78.3)%		9.8 (2.6-36.7)%		47.2 (29.7-75.0)%	
>14 years	72	52.3 (40.5-64.1)%		55.8 (43.7-67.6)%		10.4 (5.1-21.0)%		42.8 (32.3-56.8)%	
**BCR::ABL1**			0.095		0.078		0.956		0.182
Positive	21	41.9 (21.9-63.5)%		47.1 (26.5-68.3)%		10.0 (2.7-37.3)%		48.1 (30.7-75.3)%	
Negative	74	52.1 (39.4-64.7)%		58.1 (45.4-70.2)%		10.6 (5.2-21.6)%		42.0 (31.1-56.5)%	
**Time relapse after first HSCT**			0.615		0.969		0.877		0.703
≤180 day	19	45.1 (22.9-68.4)%		56.2 (33.1-77.9)%		9.1 (2.4-34.1)%		46.8 (29.6-74.1)%	
>180 day	73	52.2 (39.4-64.8)%		56.4 (43.5-68.8)%		11.1 (5.4-22.6)%		42.3 (31.3-57.2)%	
**Time between first and second HSCT**			0.882		0.907		0.619		0.800
≤365day	28	48.5 (29.2-68.5)%		51.9 (32.0-71.6)%		8.1 (2.2-30.6)%		48.1 (31.5-73.4)%	
>365 day	67	49.9 (36.4-63.3)%		56.3 (42.9-69.3)%		11.5 (5.7-23.4)%		42.2 (30.8-57.8)%	
**Intervals from CAR-T to second HSCT**			0.001		0.003		0.342		0.030
≤90days	84	53.9 (41.8-65.8)%		58.9 (46.8-70.4)%		9.5 (4.6-19.4)%		41.2 (30.8-55.0)%	
>90 days	11	18.2 (2.1-45.1)%		27.3 (6.3-55.9)%		18.2 (5.2-63.7)%		63.6 (40.7-99.5)%	
**Intervals from CAR-T to second HSCT**			0.319		0.550		0.755		0.662
≤60days	38	56.9 (39.7-73.3)%		58.5 (40.8-75.2)%		9.2 (3.1-27.3)%		41.5 (27.2-63.3)%	
>60 days	57	45.7 (31.8-60.0)%		53.5 (39.6-67.2)%		11.5 (5.3-24.7)%		44.7 (32.7-61.1)%	
**Type of CAR-T**			0.051		0.221		0.307		0.435
Auto	57	60.2 (46.1-73.4)%		62.0 (48.0-74.9)%		7.4 (2.8-19.5)%		38.3 (27.5-53.4)%	
Allo	28	32.7 (14.2-54.4)%		44.8 (24.7-65.8)%		17.3 (7.8-38.5)%		54.4 (37.7-78.5)%	
**CRS after CAR-T**			0.827		0.555		0.206		0.718
Yes	56	53.4 (39.9-66.7)%		60.0 (46.2-73.0)%		13.4 (6.7-26.9)%		40.0 (28.5-56.2)%	
No	31	44.5 (25.0-64.9)%		49.7 (30.2-69.2)%		6.2 (1.6-24.3)%		47.0 (32.8-67.3)%	
**MRD status before second HSCT**			0.612		0.817		0.354		0.862
MRD-negative	86	48.5 (36.9-60.1)%		54.5 (42.9-65.9)%		11.4 (6.1-21.3)%		44.3 (34.1-57.5)%	
MRD-positive	9	66.7 (34.6-91.9)%		66.7 (34.6-91.9)%		0		33.3 (13.2-84.0)%	
**Conditioning regimens**			0.925		0.686		0.989		0.879
TBI-based	75	52.3 (40.5-64.0)%		54.3 (42.3-66.1)%		10.3 (5.0-21.0)%		44.4 (33.9-58.1)%	
Bu-based (482day)	19	43.4 (16.6-72.5)%		54.7 (26.4-81.5)%		10.5 (2.8-39.1)%		41.7 (21.9-79.2)%	
**Type of ATG**			0.066		0.233		0.011		0.635
ATG-F	52	65.7 (51.4-78.7)%		65.7 (51.4-78.7)%		2.0 (0.3-13.7)%		38.2 (26.2-55.7)%	
ATG-T	36	39.4 (23.5-56.4)%		44.5 (28.0-61.7)%		13.8 (6.1-31.2)%		52.7 (38.0-73.2)%	
ATG-P	7	28.6 (3.7-64.7)%		71.4 (35.3-96.3)%		42.9 (18.2-100.0)%		28.6 (8.9-92.2)%	
**Second HSCT donor type, n (%)**			0.755		0.707		0.533		0.492
Matched sibling/unrelated donor	27	55.8 (35.6-75.0)%		57.3 (36.1-77.2)%		11.1 (3.8-32.3)%		39.0 (22.8-66.7)%	
Haploidentical donor	68	48.7 (36.1-61.4)%		54.9 (42.1-67.3)%		9.6 (4.5-20.6)%		45.2 (34.1-59.8)%	

HSCT, hematopoietic stem cell transplantation; CAR-T, chimeric antigen receptor T cell; CR, complete remission; LFS, leukemia-free survival; OS, overall survival; RI, relapse incidence; NRM, non-relapse mortality; CRS, cytokine release syndrome; MRD, minimal residual disease; TBI, total body radiation; Bu, busulfan; ATG-T, ATG-thymoglobuline; ATG-F, ATG-Fresenius; ATG-P, ATG-porcine; MSD-HSCT, matched sibling donor hematopoietic stem cell transplantation; Haplo-HSCT, haploidentical hematopoietic stem cell transplantation; URD-HSCT, unrelated donor hematopoietic stem cell transplantation; 95%CI, 95% confidence interval.

### Multivariate analysis

Multivariable analysis confirmed that interval from CAR-T to second HSCT >90 days (HR, 4.10; 95%CI,1.64-10.24; *p*=0.003) and ATG-T (HR, 2.83; 95%CI, 2.83-1.37; *p*=0.005) were associated with an inferior LFS. Interval from CAR-T to second HSCT >90 days (HR, 2.67; 95% CI, 1.24-5.74; *p*=0.012) was also a risk factor predicting a reduced OS. Interval from CAR-T to second HSCT >90 days (HR,2.45; 95%CI,1.14-5.24; *p*=0.021) significantly increased NRM (**Table 4**). Auto CAR-T (HR, 0.40; 95%CI, 0.19-0.85; *p*=0.016) was an independent factor associated with improved LFS. The use of ATG-F was associated with a lower relapse incidence (**Table 4**).

**Table 4:** Multivariate analysis of risk factors affecting leukemia-free survival (LFS), overall survival (OS), relapse incidence (RI), and non-relapse mortality (NRM).

Variables	LFS HR (95%CI)	*P* value	OS HR (95%CI)	*P* value	RI HR (95%CI)	*P* value	NRM HR (95%CI)	*P* value
BCR::ABL1		0.077		0.212				
Positive	1		1					
Negative	0.47 (0.20-1.08)		0.64 (0.32-1.29)					
Intervals from CAR-T to second HSCT		0.003		0.012				0.021
≤90days	1		1				1	
>90 days	4.10 (1.64-10.24)		2.67 (1.24-5.74)				2.45 (1.14-5.24)	
Type of CAR-T		0.016						
Allo	1							
Auto	0.40 (0.19-0.85)							
Type of ATG								
ATG-F	1				1			
ATG-T	2.83 (2.83-1.37)	0.005			1.42E+06 (5.08E+05-3.99E+06)	<0.001		
ATG-P	1.79 (1.79-0.55)	0.334			5.66E+06 (2.06E+06-1.56E+07)	<0.001		

CAR-T chimeric antigen receptor T cell, LFS leukemia-free survival, OS overall survival, RI relapse incidence, NRM non-relapse mortality, HSCT hematopoietic stem cell transplantation, Allo allogeneic, Auto autologous, ATG-F ATG-Fresenius, ATG-T ATG-thymoglobulin, ATG-P ATG- porcine, 95%CI 95% confidence interval.

### Other treatments after the second HSCT

Six patients became MRD-positive at a median time of 201 (range: 33 to 1098) days after the second transplant. Three patients received CD19 or CD22 CAR-T cell therapy, one with anti-CD22 monoclonal antibody (inotuzumab ozogamicin), one with anti-PD-1 monoclonal antibody (camrelizumab), and one received DLI, and all patients turned MRD-negative.

A total of 9 patients received DLI after the second transplant and the reasons were: prevention of relapse (n=5), mixed chimerism (n=1), treatment of PTLD (n=1), graft dysfunction (n=1), and treatment of MRD positivity (n=1). The median time to DLI was 102 days (range:17 to 521) after the second transplant.

## Discussion

Relapse remains one of the primary causes of treatment failure in allo-HSCT for B-ALL. CAR-T cell therapy emerged as a promising approach for patients who relapsed post-transplant. However, the majority of patients struggled to maintain long-term remission (11, 23, 24). The duration of remission for B-ALL patients who relapsed after allo-HSCT treated with donor-derived anti-CD19 CAR-T cells was only 1-22 months and the overall survival time was only 2-22 months (16, 24–26). Long-term follow-up data from EBMT showed that 120 ALL patients who relapsed after their first transplant received a second transplant with an expected 10-year OS of 5 ± 3%, LFS of 4 ± 2%, RI of 60 ± 5% and NRM of 36 ± 5% (27). A retrospective analysis of 199 second transplants of hematologic malignancy (48.2% B-ALL) from our center showed that achieving MRD-negative CR, HCT-CI score of 0 before allo-HSCT2, and utilizing a new mismatched haplotype donor were predictive factors of improved OS and LFS compared to patients without these characteristics in second transplant (28). The 2-year OS was 56.5% for the MRD-negative and 22.2% for the MRD-positive/NR groups, respectively (28). Consequently, achieving CR or even MRD-negative CR is crucial before undergoing a second transplant. B-ALL patients who relapsed after the first transplant and achieved CR with CAR-T therapy, followed by consolidation second allo-HSCT, maybe associated with a long survival. There have been limited studies with relatively small sample that analyzed the long-term survival of CAR-T followed by second HSCT for B-ALL (12, 15) In this study, which enrolled the largest sample to our knowledge, we retrospectively analyzed 95 B-ALL patients who relapsed after first transplant and received CAR-T therapy followed by consolidation second allo-HSCT to investigate whether such a treatment sequence can improve the long-term survival and to explore the risk factors associated with the prognosis.

Our results showed that patients could significantly benefit from CAR-T therapy followed by consolidation second transplant. After the second transplant, the 1-year OS and LFS were as high as 69.3% and 63.9%, respectively. The 3-year OS and LFS could also reach 55.3% and 49.8%, respectively. A retrospective study from EBMT showed that 2632 patients with hematological malignancies who underwent a second transplant for relapse of the primary transplant had a survival rate of 40% and 20% at 1 and 5 years respectively. The cumulative incidence of NRM was 33% at 1 year and 40% at 5 years. The incidences of relapse were 36% and 45%, respectively (29). In our study cohorts, the improvement in OS compared to historical data was mainly due to a decrease in post-transplant relapse rate. CAR-T therapy may enable more patients to achieve CR or even MRD-negative CR, providing them with the opportunity to bridge into a second transplant. When deep remission is achieved after CAR-T cell therapy, it is possible to reduce the relapse rate after second transplant. DLI followed by MRD test and GVHD-guided multiple DLIs decreased relapse and increased survival post-transplant in patients with R/R acute leukemia who received allo-HSCT (30). In our study, a proportion of patients used prophylactic DLI after the second transplant, which maybe another contributing factor to the low cumulative relapse rate after second HSCT.

In this study, the 100-day incidence of grade 2 to 4 and grade 3 to 4 aGVHD was 43.2% and 8.4%, respectively, which was similar to a previously published parallel comparative study from our center showing that the incidence of grade 2-4 and grade 3-4 aGVHD was 48.1% and 11.1% for B-ALL patients who received first allo-HSCT after achieving CR with CAR-T therapy (31). In addition, the 100-day cumulative incidence of grade 3-4 aGVHD in our study was less than 10%, which is similar to other previously reported rates in first allo-HSCT without receiving CAR-T therapy (32, 33). However, the grade 3 to 4 aGVHD was lower than our previously reported in second transplant for hematological malignancies such as acute myeloid leukemia (AML), myelodysplastic syndrome (MDS), and chronic myeloid leukemia (CML)(8.4% vs 15.1%) (28). In our previous report, 21.1% of patients were in an advanced stage before second transplant who may receive high intensity myeloablative conditioning (MAC) regimens (28). The incidence of extensive cGVHD after second transplant was 33.1% and 22% in our and Johns Hopkins studies, respectively (34). In the Johns Hopkins study, most patients received a reduced intensity conditioning (RIC) regimen and GVHD prophylaxis included post-transplant cyclophosphamide (PTCy) in 83% of cases (34). The intensity of the conditioning regimens was associated with the incidence of cGVHD (28, 34, 35). Grade 3-4 aGVHD and moderate/severe cGVHD were more common in the MAC group than in RIC group (36). In our study, all patients were in CR before the second transplant and received the MAC allo-HSCT, which may partially explain the different occurrence of GVHD between our center and the other centers.

The NRM in the EBMT study was 32 ± 3%, 33 ± 3% and 35 ± 3% at 2, 5 and 10 years, respectively (27). In our study, the 1-year and 3-year NRM was 30.7% and 43.6%, respectively. A retrospective analysis of 71 patients who received a second HSCT using reduce intensity conditioning (RIC) regimens after disease relapse following an initial allo-HSCT revealed that the predicted OS and TRM at 2 years were 28 and 27%, respectively (37). EBMT data demonstrated that NRM was significantly lower when RIC was administrated in the second transplant (29). Although our study indicated that some patients achieved long-term survival with CAR-T cell therapy followed by a second transplant, the low RI and high NRM suggest the need to reduce the conditioning regimen intensity of second transplant to improve survival. CAR-T therapy enables deeper remissions, creating the conditions conducive to a reduced intensity conditioning regimen, which may help lower the incidence of NRM. In this study, TA-TMA was one of the top three causes of death. The cumulative incidence of TA-TMA was 8.4% at 100 days, 14.8% at 1 year and 19.5% at 3 years after second HSCT, which were much higher than the incidence of TA-TMA reported in the literature (38). Second HSCT is a risk factor for the incidence of TA-TMA (39, 40). All patients in this study had received multiple lines of treatment, resulting in vascular endothelium damage. Two cases of TMA following CAR-T treatment have been reported (41). Whether CAR-T itself increases the incidence of TA-TMA requires further investigation through controlled studies.

In second transplantation, disease status of MRD-negative CR, HCT-CI score of 0 prior to allogeneic HSCT, and new mismatched haplotype donors, an interval from first transplant to relapse >10 months and TBI as part of the conditioning for second transplant were predictors of improved OS and LFS compared to patients without these characteristics (27, 28, 34). In those studies, the effect of CAR-T treatment on second transplant outcomes was not analyzed, although there were some cases of bridging to a second transplant after CAR-T. Multivariable analysis in our study confirmed that interval from CAR-T to second HSCT >90 days were associated with an inferior LFS(HR, 4.10; 95%CI,1.64-10.24; *p*=0.003) and OS(HR, 2.67; 95% CI, 1.24-5.74; *p*=0.012). The time interval between CAR-T and second HSCT >90 days is a high-risk factor for NRM. In the >90 days group, the incidence of TA-TMA was higher than in the ≤90 days group. In fact, patients with poor conditions often miss the opportunity to undergo transplant, which may also explain why these patients are more likely to develop TA-TMA. Auto CAR-T (HR, 0.40; 95%CI, 0.19-0.85; *p*=0.016) was an independent factor associated with improved LFS. Obtaining CAR-T cells depend on the quality of leukapheresis. The ability to collect sufficient numbers of lymphocytes for CAR-T cell production often predicts that the patient has less than 30% peripheral blood tumor cells. The use of ATG-F was associated with a lower relapse incidence. The possible reason is that the metabolism of active ATG-T is slower than that of active ATG-F, which has a stronger affinity for the antigen and is therefore more immunosuppressive (42, 43). In allo-HSCT for severe aplastic anemia, ATG-F and ATG-P or ATG-T and ATG-P in conditioning regimen did not differ significantly in terms of OS, grade 3 to 4 aGVHD and moderate to severe cGVHD (44, 45). However, no control studies have been performed on the pharmacokinetics of ATG-P and ATG-T or ATG-P and ATG-F, and the reason for the high relapse rate in the ATG-P group needs to be explored. On the other hand, with only 7 patients in the ATG-P group, the conclusion of an increased relapse rate in the ATG-P group needs to be verified by enrolling more patients.

Age was not a prognostic factor in this study. The conclusions are similar to another study from Johns Hopkins (34). The median age of patients was 22.8 (3.3-52.8) years in our study and 43.9 (1–74) years in the Johns Hopkins study. There were no significant differences in OS, LFS, NRM and RI between the MRD-positive and MRD-negative groups in our study. Moreover, 66.7% (6/9) of MRD-positive patients and 15.1% (13/86) of MRD-negative patients received intensified conditioning regimens (*p*<0.001). Intensified conditioning regimens may help to reduce post-transplant RI and improve survival.

Some of the patients were treated with CAR-T at other institutions. The effect of the doses of CAR-T cells on transplant outcomes could not be detailed analyzed. The dose of CAR-T cells was considered to be an important factor (46). This study is limited by its retrospective nature and the small sample size. The lack of a control group also limits the ability to draw definitive conclusions about the efficacy of the treatment sequence. Overall, while the study provides valuable insights into the potential benefits of a consolidation second transplant after CAR-T therapy in B-ALL patients who relapsed after a first transplant, there are several limitations that need to be considered. Future studies with larger sample sizes, longer follow-up periods, and control groups are needed to confirm these findings.

In conclusion, our study shows that CAR-T therapy followed by consolidation second transplant could significantly improve the long-term survival in B-ALL patients who relapsed after a first HSCT. The second transplant could be considered in suitable patients and is recommended to perform within 90 days after CAR-T treatment. Reducing the intensity of the conditioning regimen needs to be explored to reduce the NRM.

## Data availability statement

The original contributions presented in the study are included in the article/**Supplementary Material**. Further inquiries can be directed to the corresponding authors.

## Ethics statement

The studies involving human participants were reviewed and approved by the Ethics Committee of Hebei Yanda Lu Daopei Hospital. Written informed consent to participate in this study was provided by the participants’ legal guardian/next of kin.

## Author contributions

X-YC developed the concept and designed the study. X-YC, J-PZ, Y-LZ, MX, J-RZ, YL, R-JS, Z-JW and D-YL performed the transplant for patients. XZ, J-FY and PL conducted the clinical trials of CAR-T. X-YC made the major contributions to the analysis of clinical data. X-YC drafted and wrote the manuscript. PL revised the manuscript. All authors contributed to the article and approved the submitted version.
