# Supplementary material for: Analysis benefits of a second Allo-HSCT after CAR-T cell therapy in patients with relapsed/refractory B-cell acute lymphoblastic leukemia who relapsed after transplant
Source: Front Immunol. 2023 Jul 4;14:1191382. doi: 10.3389/fimmu.2023.1191382 (PMC10352576; doi:10.3389/fimmu.2023.1191382)
Supplement: Supplementary file 1 [file Table_1.doc]

**Table S1. Treatment received after MRD-positive or relapse after the first transplantation**

|  | Treatment after MRD-positive  (n=34) | Treatment after relapse  (n=92) |
| --- | --- | --- |
| TKI | 9(26.5%) | - |
| Reduced immunosuppressive dose | 8(23.5%) | - |
| TKI-DLI | 4(11.8%) | - |
| Chemotherapy-DLI | 4(11.8%) | - |
| CAR-T | 3(8.8%) | 8(8.7%) |
| Interferon | 3(8.8%) | - |
| Chemotherapy | 2(5.9%) | - |
| Chemotherapy-CAR-T | 1(2.9%) | 56(60.9%) |
| Chemotherapy-TKI-CAR-T | - | 8(8.7%) |
| Chemotherapy-DLI-CAR-T | - | 5(5.4%) |
| Chemotherapy-TKI-DLI-CAR-T | - | 2(2.2%) |
| Radiotherapy-chemotherapy-CAR-T | - | 2(2.2%) |
| CAR-T-DLI-CART | - | 1(1.1%) |
| CAR-T-DLI-chemotherapy-CAR-T | - | 1(1.1%) |
| CAR-T-chemotherapy-CART | - | 1(1.1%) |
| CAR-T-radiotherapy-PD1 antibody-chemotherapy-CAR-T | - | 1(1.1%) |
| Daratumumab-CAR-T | - | 1(1.1%) |
| Chemotherapy-CAR-T-chemotherapy-CAR-T | - | 1(1.1%) |
| Chemotherapy-CAR-T-TKI-CAR-T | - | 1(1.1%) |
| Chemotherapy-DLI -radiotherapy-CAR-T | - | 1(1.1%) |
| Chemotherapy- radiotherapy-CAR-T | - | 1(1.1%) |
| Chemotherapy-CAR-T+NK-IL-2-CAR-T | - | 1(1.1%) |
| TKI-CAR-T | - | 1(1.1%) |

# CAR-T chimeric antigen receptor T cell, TKI tyrosine kinase inhibitor, DLI donor lymphocyte infusion, MRD minimal residual disease.

**Table S2. Characteristics and outcomes of patients in the groups of ≤90 days and >**90 days between the last CAR-T infusion and the second transplant.

| Variables | ≤90 days group  (n=84) | >90 days group  (n=11) | *p* value |
| --- | --- | --- | --- |
| Age, median(range)years at second HSCT | 22.5(3.3-52.8) | 26.2(7.2-49.0) | 0.632 |
| Time between first and second transplant, median(range)days | 500(160-2813) | 625(245-4377) | 0.185 |
| CAR-T as first-line treatment |  |  | 0.467 |
| Yes | 9 | 2 |  |
| No | 75 | 9 |  |
| Gender, male/female | 49/35 | 7/4 | 0.737 |
| MRD status before the second transplant; n |  |  | 0.254 |
| MRD-negative | 75 | 11 |  |
| MRD-positive | 9 | 0 |  |
| ATG; n |  |  | 0.100 |
| ATG-F | 49 | 3 |  |
| ATG-T | 30 | 6 |  |
| ATG-P | 5 | 2 |  |
| CAR-T; n |  |  | 0.141 |
| Auto | 51 | 6 |  |
| Allo | 26 | 2 |  |
| Not available | 7 | 3 |  |
| Second HSCT donor type; n |  |  | 0.535 |
| Matched sibling/unrelated donor | 23 | 4 |  |
| Haploidentical donor | 61 | 7 |  |
| Stem cell source; n |  |  | 0.535 |
| BM+PB | 61 | 7 |  |
| PB | 23 | 4 |  |
| Infused MNC count; median(range)×10^8^/kg | 9.1(8.0-11.3) | 8.6(7.9-9.7) | 0.212 |
| Infused CD34^+^ cells count; median(range)×10^6^/kg | 5.0(4.0-5.7) | 5.0(4.5-5.1) | 0.820 |
| Infused CD3^+^ cells count; median(range)×10^8^/kg | 1.9(1.7-2.1) | 1.7(1.4-1.9) | 0.575 |
| DLI after HSCT; n |  |  | 0.254 |
| Yes | 9 | 0 |  |
| No | 75 | 11 |  |
| Neutrophil engraftment at day 30; (95%CI) | 100.0 (100.0-100.0)% | 100.0 (100.0-100.0)% | 0.608 |
| Platelet engraftment at day 30;(95%CI) | 89.3(82.9-96.2)% | 81.8 (61.9-100.0)% | 0.894 |
| 2-4 grade aGVHD at day 100;(95%CI) | 41.7(32.35-53.7)% | 54.6(31.8-93.6)% | 0.430 |
| 3-4 grade aGVHD at day 100;(95%CI) | 8.3 (4.1-16.9)% | 9.1(1.4-58.9)% | 0.971 |
| All cGVHD at day180;(95%CI) | 28.9 (20.6-40.5)% | 36.4(16.6-79.5)% | 0.709 |
| Extensive cGVHD at day180;(95%CI) | 24.1 (16.5-35.3)% | 27.3(10.4-71.6)% | 0.700 |
| CMV viremia at day 100;(95%CI) | 73.8 (65.0-83.8)% | 63.6 (40.7-99.5)% | 0.697 |
| EBV viremia at 100 day;(95%CI) | 19.1(12.3-29.6)% | 18.2(5.2-63.7)% | 0.928 |
| TA-TMA at 1 year; (95%CI) | 10.7(5.8-19.9)% | 45.5(23.8-86.8)% | 0.018 |
| Death due to infection at 3 year;(95%CI) | 19.0 (12.0-30.2)% | 9.1(1.4-58.9)% | 0.454 |

MRD Minimal residual disease, ATG-F ATG-fresenius, ATG-T ATG-thymoglobuline, ATG-P ATG-porcine, CAR-T chimeric antigen receptor T cell, BM bone marrow, PB peripheral blood, MNC mononuclear cells, DLI donor lymphocyte infusion, aGVHD acute graft-versus-host disease, cGVHD chronic graft-versus-host disease, TA-TMA transplant-associated thrombotic microangiopathy, 95%CI 95% confidence interval.
